# Dr. Rochester’s Successor

**Published:** 1887-08

**Authors:** 


### Editorial.

### *DOCTOR ROCHESTER'S SUCCESSOR.*

It gives us great pleasure to announce that the Faculty of the Buffalo Medical College have elected Dr. Chas. G. Stockton to the Chair of Theory and Practice of Medicine and Clinical Medicine, vacated by the death of Dr. Rochester.

The alumni will see in this appointment an indication that the ruling spirits in the Buffalo Medical College are not altogether defiant to the demands which its true friends have made, and which culminated in the action of the Erie County Society at its last meeting. These demands, as repeatedly expressed in the JOURNAL, may be summarized as follows:

1st. That the college should cease to confer the degree of M. D. upon unqualified men.

2d. That, other things being equal, its own alumni should be selected to fill vacancies.

3d. That the professional talent of our own city should be recognized, and the importation of men, unless of exceptional ability, from other cities discountenanced.

In the appointment of Dr. Stockton, the last two of these requirements have been fulfilled. Dr. Stockton is a graduate of the Buffalo Medical College, and since his graduation has made Buffalo his home.

In 1882, he, together with other prominent men who despaired in any other way of effecting a reform in the loose methods then profitably followed by those who controlled the medical education of Western New York, effected the establishment of a medical department of Niagara University, and was by the trustees appointed to the Chair of Materia Medica and Therapeutics, which position he has held with honor to himself and the University to the present time.

That such a man, irrevocably committed to the cause of medical educational reform, should have been elected to the most important chair in the Buffalo Medical College, is a most gratifying indication that the days of easily-obtained diplomas are at an end in Buffalo. Knowing the honesty of Dr. Stockton's convictions, we are confident that, without such assurances, he could not have been prevailed upon to accept the proffered chair.

The alumni of the old college will, therefore, rejoice with us in the hope that it will follow in the steps of Niagara University and take its place among the most reputable colleges in the land.

We extend, therefore, our most hearty congratulations upon this appointment to the Buffalo Medical College, and to the worthy alumni of the college, with the earnest hope that every successive vacancy may be as well and honorably filled.

